# Supplementary material for: The interactome of intact mitochondria by cross-linking mass spectrometry provides evidence for coexisting respiratory supercomplexes
Source: Mol Cell Proteomics. 2017 Dec 8;17(2):216–32. doi: 10.1074/mcp.RA117.000470 (PMC5795388; doi:10.1074/mcp.RA117.000470)
Supplement: Supplemental Data [file supp_RA117.000470_133922_0_supp_23978_fzffwf.zip › spectra_annotation/mito_spectra_annotation/416-1-1-1-124-1.pdf]

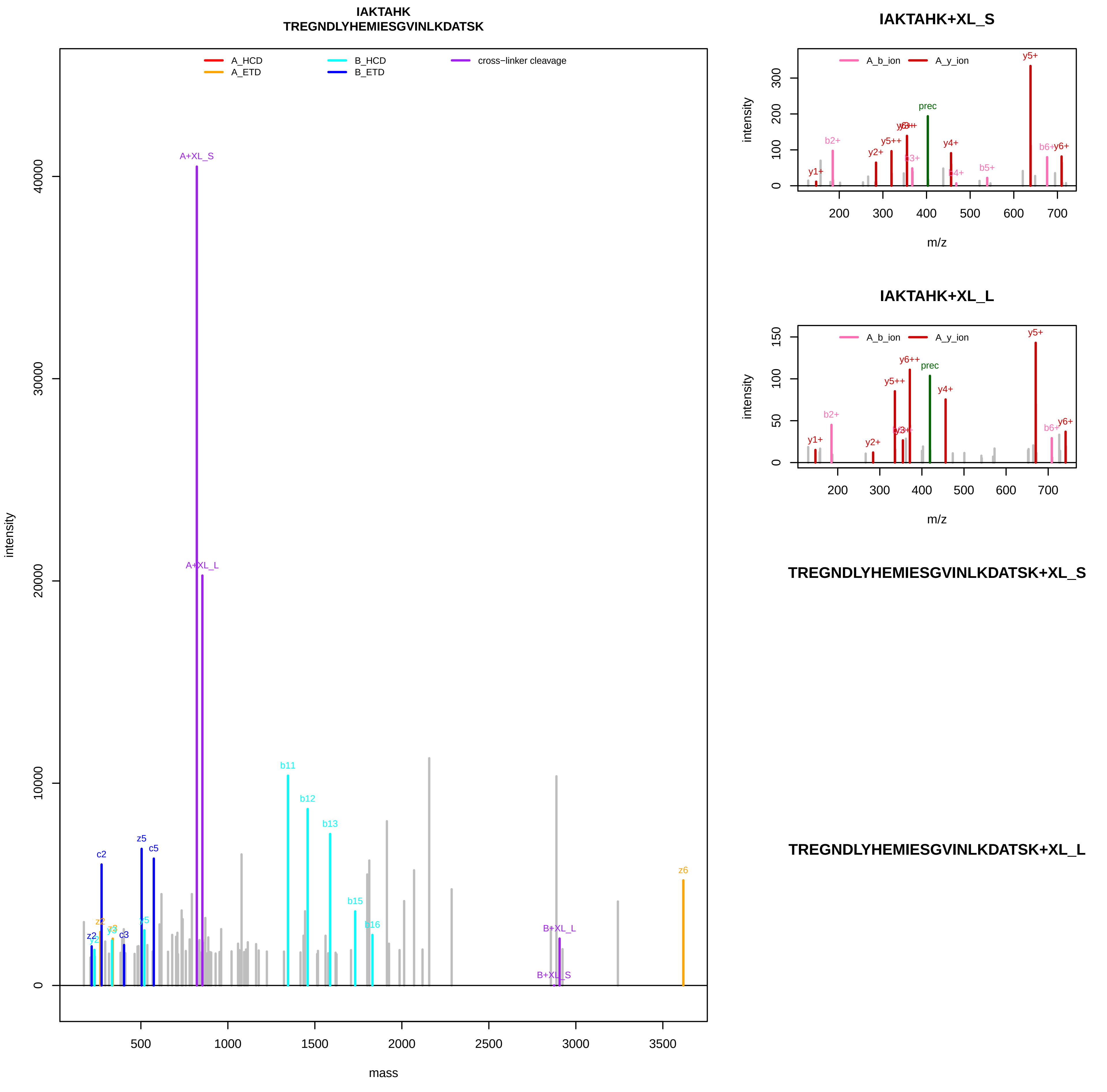
IAKTAHK
TREGNDLYHEMIESGVINLKDATSK
A_HCD
A_ETD
B_HCD
B_ETD
cross−linker cleavage
A+XL_S
A+XL_L
B+XL_L
B+XL_S
b11
b12
b13
b15
b16
z2
y2
z2
z3
y3
c3
c2
z5
c5
y5
z6
intensity
mass
0
10000
20000
30000
40000
500
1000
1500
2000
2500
3000
3500
IAKTAHK+XL_S
A_b_ion
A_y_ion
prec
y1+
b2+
y2+
y5++
y6++
b3+
y4+
b4+
b5+
y5+
b6+
y6+
intensity
m/z
0
100
200
300
200
300
400
500
600
700
IAKTAHK+XL_L
A_b_ion
A_y_ion
prec
y1+
b2+
y2+
y5++
y6++
y3+
y4+
y5+
b6+
y6+
intensity
m/z
0
50
100
150
200
300
400
500
600
700
TREGNDLYHEMIESGVINLKDATSK+XL_S
TREGNDLYHEMIESGVINLKDATSK+XL_L
